# A systematic review and meta‐analysis of mental health service use in people who report psychotic experiences

**DOI:** 10.1111/eip.12464

**Published:** 2017-08-14

**Authors:** Vishal Bhavsar, Philip McGuire, James MacCabe, Dominic Oliver, Paolo Fusar‐Poli

**Affiliations:** ^1^ Department of Psychosis Studies, Institute of Psychiatry, Psychology and Neuroscience King's College London UK

**Keywords:** confounding, epidemiology, health services, self‐reported psychotic experiences, systematic review

## Abstract

**Background:**

Self‐reported psychotic experiences (PEs) are associated with psychopathology of all kinds, not just psychoses. However, systematic reviews on the relevance of this for health services are unavailable. Furthermore, whether association with service use is confounded by other psychopathology is unknown, and is relevant to prevention and treatment.

**Objectives:**

Literature examining associations between PEs and service use was systematically reviewed. Study quality and the direction and extent of any associations were assessed, and meta‐analysis conducted.

**Methods:**

Systematic review and meta‐analysis was carried out as per PRISMA guidelines. A search of electronic databases was performed based on free‐text and structured terms. Included studies were evaluated by two raters using a structured tool and estimates extracted for reporting.

**Results:**

Thirteen studies were returned. We found two prospective studies, and a minority of studies accounted for concurrent psychopathology, limiting our ability to test our main hypotheses. Five studies reported associations by different types of service use. Almost all studies assessed service use by self‐report. Meta‐analysis suggested that people who reported PEs were around twice as likely to report service use compared to those who did not (pooled OR for all included studies: 2.20,95% confidence intervals (95%CI): 1.66,2.91).

**Conclusions:**

There was consistent evidence of association between PEs and mental health service use at the general population level. However, evidence for causation was poor due to a limited number of studies. Whether increased service use in this group is solely attributable to PEs, and therefore whether interventions aimed at limiting/preventing PEs might be effective, requires studies focusing on the relationships between PEs, psychopathology and service use.

## 1 INTRODUCTION

### 1.1 Rationale for systematic review

The diagnosis of psychotic disorder under ICD‐10 and DSM‐V depends primarily on the presence of clinically‐ascertained psychotic symptoms for a specified length of time, together with other features such as loss of function and disorganized behaviour (Association AP, 2013; World Health Organization, 1992). The presence of self‐reported psychotic‐like experiences (PEs), which do not reach the threshold for a diagnosable psychotic disorder, may be associated with an elevated risk for developing psychotic disorders in the future (Fusar‐Poli et al., 2013). However, limited evidence also suggests that PEs are associated with impacts on mood, functioning, and suicidality, indicating that these symptoms might carry wider implications for public mental health than for the development of psychosis alone (Armando et al., 2012; Fusar‐Poli et al., 2014; Kelleher et al., 2012a; Kelleher et al., 2014a; Yung et al., 2006). This leads us to hypothesise that people with PEs could be greater consumers of mental health services, including primary care, compared to those without (DeVylder et al., 2014).

Nevertheless, it is unclear whether PEs themselves cause service use, or whether this relationship is confounded by concurrent psychopathology or suicidal thinking and behaviours. This has implications for treatment ‐ if PEs are a cause of service use, then these symptoms should be the focus for intervention, however, if the association is confounded by other psychopathology then PEs should trigger assessment and treatment of concurrent psychopathological symptoms. This is the subject of the present systematic review.

### 1.2 Aims and Objectives

The aim of this systematic review was to consolidate and summarise research findings on mental health service use of individuals reporting PEs.

The objectives were to:

- to summarise qualitatively, and quantitatively if possible, the association between PEs and service use, and
- to assess alternative explanations for any associations found, by evaluating whether studies attempted to account for confounding by non‐psychotic psychopathology.

## 2 METHODS

### 2.1 Selection Procedures

PRISMA (Moher et al., 2009) guidelines were adhered to throughout (see Supplementary material for the PRISMA checklist for this review). The review gathered general population‐based studies that ascertained the presence of self‐reported PEs and evaluated mental health service use. Mental health service use was defined as the use of health services for any mental health problem, and so included use of primary care. For the purposes of this review, PEs were defined as phenomena observed in people with psychosis (such as hallucinations, persecutory ideation, thought interference phenomena, and unusual experiences) reported by subjects in general population samples, i.e. in samples not only selected on the basis of using mental health care.

A systematic search strategy was employed to select relevant studies for the review. Search terms referring to PEs and to service use for mental health problems were combined with terms identifying observational epidemiological studies. We included search terms used in previous reviews in the PE literature (Kaymaz et al., 2012; Kelleher et al., 2012b; Linscott & van Os, 2013a). Electronic databases (EMBASE, MedLINE, and PsychInfo) were searched since their inception for the relevant search terms, on January 1^st^ 2017. Search terms referring to PEs and to service use for mental health problems were combined with terms identifying observational epidemiological studies. The full list of search terms is presented in an Appendix S1, supporting information, to this paper. We also searched reference lists for three meta‐analyses of PEs published since 2010 (Kaymaz et al., 2012; Kelleher et al., 2012b; Linscott & van Os, 2013a). Reference lists for returned studies were searched for additional articles.

### 2.2 Selection Criteria

Both cross‐sectional studies and follow‐up studies were considered acceptable. We placed no restriction on year of publication. Candidate studies from the published scientific literature were selected using a combination of inclusion and exclusion criteria (see Table 1). Searches were limited to full text articles in English. Titles and abstracts were screened for all articles initially returned from the search terms.

**Table 1:** Inclusion and exclusion criteria for systematic review

Inclusion criteria	Observational studies adopting either a case control, cohort or cross‐sectional design
**Exclusion Criteria**	Participants not sampled from the general population of the study area
	Intervention studies
	Studies not written in English
	Not peer reviewed
	Conference abstracts

### 2.3 Data extraction

Studies meeting systematic review criteria were subject to data extraction using a form containing fields for study centre, type of geographic location, sample size, population details, duration of study, study design type, exposure, outcome measures, and results. In particular, data on the size and precision of any associations found, effects of adjustments, and stratification of results by type of service use were extracted from included studies. Citation lists for the included articles, and for the three pre‐existing meta‐analyses of PEs (Kaymaz et al., 2012; Kelleher et al., 2012b; Linscott & van Os, 2013b) were searched in order to identify further potential studies.

### 2.4 Quality assessment

Evaluation of study quality on all included studies was done separately by two raters (V.B. and D.O.) using a risk of bias tool, the Newcastle Ottawa Scale (Wells et al., 2000) with information collected on case definition and its validity, representativeness of cases, definition and selection of controls, comparability of cases and controls (including control for confounding), ascertainment of exposure, and description of non‐response. Results on this tool were used to derive a quality score for each study that was tabulated, along with details for each field. The primary summary measure of interest was the effect size comparing mental health service use in people reporting PEs to that in those not reporting PEs.

Although there were a small number of returned studies with considerable heterogeneity, meta‐analysis was nevertheless attempted in order to provide a quantitative summary of the association of interest (see discussion section).

### 2.5 Statistical analysis/synthesis of results

The principal summary measure for this systematic review was the odds ratio (OR). Data were entered into an electronic database and analysed quantitatively using Comprehensive Meta‐Analysis Software, version 3 (Inc. B, 2014). This software allowed the meta‐analysis of effect sizes using a combination of counts for each group (PEs and service use, PEs and no service use, no PEs and service use, and no PEs and no service use) and study estimates, where counts were not available, with different estimates being weighted in the final model according to the inverse variance method.

Heterogeneity among estimates was evaluated using the *Q* statistic, with the magnitude of heterogeneity measured with the *I* ^*2*^ index (DerSimonian & Laird, 1986). Given high probable heterogeneity among the studies, random effects meta‐analytic models were used throughout.

### 2.6 Risk of bias across studies

Risk of publication bias was evaluated by visual inspection of funnel plots and application of the Egger regression intercept method (Egger et al., 1997).

### 2.7 Additional analyses

Analyses were repeated with each study removed in turn, to assess sensitivity of our inferences to the exclusion of different studies. The classic fail‐safe N method (Rothstein et al., 2006) was used to estimate the number of unpublished studies that would be required in order bring the meta‐analytic p value for the association to a level greater than the pre‐defined alpha level of 0.05.

## 3 RESULTS

### 3.1 Retrieved studies

**Figure 1:**
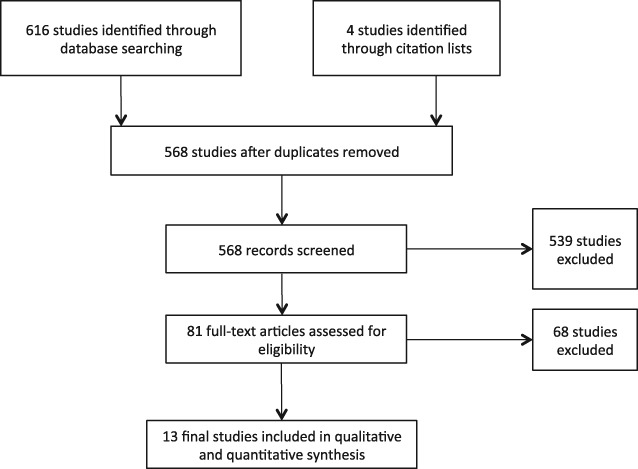
Study selection: flow of information through the systematic review process.

The flow of articles through the study, including numbers of articles screened, assessed for eligibility and included in the review, is summarised in the diagram displayed in Figure 1. For each included study, characteristics for which data were extracted, including study, follow‐up period, and exposure/outcome assessment, are summarised in Table 2, together with information on risk of bias and summary results.

**Figure 2:**
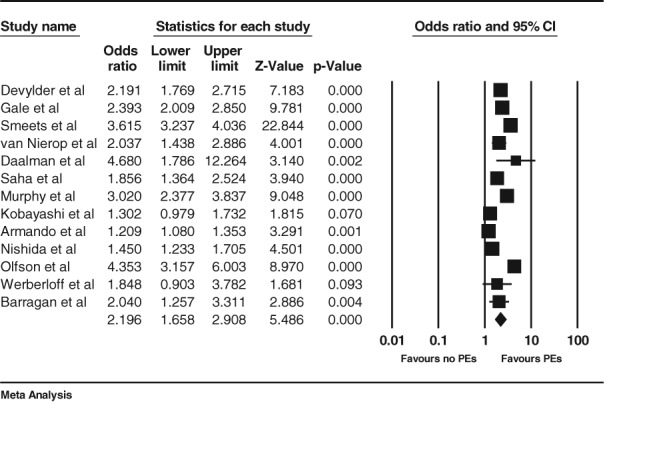
Estimates and forest plot from random effects meta‐analysis.

**Table 2:** Included studies in systematic review

Reference, study type, time information	Sample details	Psychotic experiences and outcome operationalization, measurement	Summary of analysis and quality notes, adjustment for confounding	Results	Notes on quality
DeVylder et al. 2014(Yung et al., 2006)	**Sample source:** Collaborative Psychiatric Epidemiology Surveys (n = 10541). **Comparison/control group:** Survey respondents who did not report PEs.	**PEs:** WHO CIDI for previous 12 months. **Outcomes:** Seven questions on use of healthcare in lifetime and the last 12 months.	Survey‐weighted regressions with adjustments for sociodemographic covariates and concurrent psychopathology, together with corrections for multiple comparisons.	Lifetime (OR 2.2, 95%CI 1.6, 3) and previous 12 months (OR 2.3, 95%CI 1.4, 3.7) care use were associated with PEs after adjustment for current affective, anxiety and substance misuse problems.	**8 stars.** Large, representative, random sample with validated exposure measures, and regression adjustment for co‐morbid psychopathology and sociodemographic variables. Self‐reported service use.
Gale et al. 2011(Rothstein et al., 2006)	**Sample source:** Representative survey of New Zealand adults (n = 7435). **Comparison/control group:** Survey respondents who did not report PEs.	**PEs:** CIDI v.3 **Outcomes:** Mental health service use was assessed using the CIDI.	Survey weighted latent class analyses were used to arrive at underlying sub‐groups of sampled subjects, defined by variables including PEs, use of different health services.	The use of hospital services, specialist mental health services in general, and any service, increased with the number of PEs. For binary latent classes, psychotic class members had a prevalence of specialist mental health service use of 68.9% (95%CI 49.8, 88.1), compared to 15.3% (95%CI 14.2, 16.4) in the normal group.	**6 stars.** Large, representative sample with good response rate. Non‐responders were not described and responders were not compared to general population. Validated measure of PEs. Self reported mental health service use. No adjustments were made for comorbid psychopathology.
Smeets et al. 2013(Daalman et al., 2016)	**Sample source:** Dutch household survey, NEMESIS‐1 (n = 7075) **Comparison/control group:** Survey participants who did not report PEs.	**PEs:** CIDI v.1.1 **Outcomes:** Respondents were asked whether they had ever having sought care from any mental health institution described in G Section of the CIDI.	Data analysis included logistic regression of accessing mental health care against PE status. Models were not adjusted for comorbid psychopathology.	Having only hallucinations (OR 2.6, 95%CI 2.1, 3.2), only delusions (OR 3.1, 95%CI 2.7, 3.7), and both hallucinations and delusions (OR 7.5, 95%CI 5.9, 9.6) were strongly associated with accessing mental health care in the lifetime.	**7 stars.** Large, representative sample, with adequate response and comparability between responders and non‐responders. Validated measurement tool for PEs. Self‐reported mental health service use. No adjustments were made for comorbid psychopathology.
Van Nierop et al. 2011, (Barragán et al., 2016)	**Sample source:** Dutch household survey, NEMESIS‐2 (n = 6646) performed 1996–1999. **Comparison/control group:** NEMESIS‐2 respondents without PEs.	**PEs:** CIDI v.3 **Outcomes:** Respondents were asked for reports of accessing mental health care in the context of any psychopathology (help from psychiatrists/ psychologists for any psychiatric problem including drug or alcohol problems) and accessing care specifically for PEs.	Analysis employed multinomial logistic regression and linear regression. No adjustments for comorbid psychopathology were made.	Compared to controls, people with false positive psychotic symptoms (i.e. with symptoms but not psychotic disorder) were nearly twice as likely to report accessing mental health care (RR 2.02, 95%CI 1.43, 2.87).	**7 stars.** Large, representative sample with adequate response; comparability described between responders and non‐responders. Validated measurement tool for measurement of PEs. Self reported mental health service use. No adjustments for comorbid psychopathology.
Armando et al. 2012(Fusar‐Poli et al., 2014), Rome, Italy, Urban.	**Sample source:** Non‐random/purposive sample of 997 college students. **Comparison/control group:** Survey respondents who did not report PEs.	**PEs:** CAPE **Outcomes:** Subjects were asked to report whether they had need to consult a psychiatrist/psychologist in the past year.	The Beck's Depression Inventory(BDI) total score was used to adjust for depression‐ ANCOVA used to assess association between accessing mental health care and factor scores for PEs, and for Beck's Anxiety Inventory and General Health Questionnaire‐12.	This study compared people accessing mental health care with non‐help seeking students on various continuous measures of different PEs and anxiety and depression/general functioning. For PEs, only important differences were found for persecutory ideation but not for perceptual abnormalities, bizarre experiences, and magical thinking.	**6 stars.** Study population sufficiently large, somewhat representative, but gathered by non‐random sampling. No description of non‐response or comparison of responders with non‐responders. Validated measurement tool for ascertainment of PEs. No control applied for comorbid psychopathology.
Daalman et al. 2016(DerSimonian & Laird, 1986), Utrecht, Netherlands, Mixed.	**Sample source:** 103 subjects with auditory/visual hallucinations (at least once per month, for at least one year) and 60 matched controls without Axis I or II disorders. **Comparison/control group:** 60 matched controls without hallucinations, or Axis I or II disorders.	**PEs:** Launay and Slade Hallucination Scale. **Outcomes:** Persistence of auditory/visual hallucinations and need for mental healthcare.	Restricted one of the regression models to people without remitted depression at baseline.	OR for total distress from auditory/visual hallucinations: 2.08 (1.107, 3.9), and 2.08 (1.002, 4.322) when depression at baseline was removed. 39.5% of people with auditory/visual hallucinations had need for mental healthcare at five year follow up, compared to 12.2% in the control group.	**5 stars.** Cases not clearly consecutive or representative of people with PEs. Community controls were selected without disorder. Restricted one of the regression models to people without depression. Cases and controls were matched, but further details on matching criteria were not reported. Mental health service use ascertained by structured interview, not blinded to hypothesis or case control status. The same method of ascertainment of service use was employed for both cases and controls. The same proportion of non‐response quoted for both groups. PEs were ascertained by self‐report, with the same tool administered to cases and to controls.
Kobayashi et al. 2011(Smeets et al., 2013),Tokyo, Japan, Urban.	**Sample source:**731 general psychiatric outpatients aged 16–30, 748 students. 2006–2008. **Comparison/control group:** 748 students from two universities and two high schools.	**PEs:** PRIME tool, a self‐report tool for screening of prodromal symptoms. **Outcomes:** Use of mental health care was distinguished by case–control status. Cases were defined as using mental health care because they were psychiatric outpatients.	Investigators carried out regression adjustment for score on the Zung Self‐Rating Depression Scale (ZSRDS).	There was no association between different PE items and utilization of mental health care after adjusting for depression. Researchers did not report adjusted associations between case–control status and the total score for PEs.	**9 stars.** Adequate case definition for mental health service use, and cases were gathered consecutively. Controls were broadly representative, but were not definitively free of service use for mental health problems. A subgroup of the participants was matched on age. One set of logistic regressions for association between individual PE items and case‐control status models was adjusted for depression score. Used a self‐report screen for PEs, and employed the same method in cases as controls. There were similar non‐participation rates in cases and controls.
Barragan et al. (Egger et al., 1997), 2015, United States of America, Mixed.	**Sample source:** Collaborative Psychiatric Epidemiology Survey, n = 11,937 **Comparison/control group:** Subjects without PEs formed the comparison group.	**PEs:** WMH‐CIDI **Outcomes:** Respondents were asked: “Have you ever in your lifetime been admitted for an overnight stay in a hospital or other facility to receive help for problems with your emotions, nerves, mental health, or your use of alcohol or drugs?” and “Which of the following types of professionals did you ever see about problems with your emotions or nerves or your use of alcohol or drugs?” Service providers were grouped into the following: informal provider (ministers, priests, spiritualist or religious advisor, herbalists, or any other healer),mental health provider (psychiatrist, psychologist, social worker, counselor, or any other mental health professional), and medical provider (general practitioner, medical doctor, nurse, occupational therapist, or any other medical professional).	No adjustment was made for concurrent psychopathology.	Association between lifetime self‐reported psychotic symptoms and use of services for mental health problems‐ OR: 2.04 (95%CI: 1.26, 3.32) for any lifetime psychotic symptom on accessing any informal/mental health provider, 2.95 (95%CI: 2.82, 4.79) for hospitalization.	**6 stars.** Large and representative sample; no description of response proportion or characteristics of non‐responders, or similarities between responders and general population. Validated measurement tool for PEs. There was no adjustment of effect for depression/other concurrent psychopathology.
Murphy et al. 2010 (Gale et al., 2011), England, Mixed.	**Sample source:** APMS 2007(n = 7266). **Comparison/control group:** APMS respondents without psychotic symptoms.	**PEs:** Psychosis Screening Questionnaire. **Outcomes:** Using mental health care in the previous year was assessed by self‐report, characterised by seeing the GP for emotional problems, seeing the GP for physical problems, and seeking counselling therapy.	Adjusted for sociodemographic barriers to referral and the presence of any neurotic disorder.	Only paranoia was significantly associated with accessing counselling/therapy after all adjustments were made (OR = 2.92 (1.54, 5.34), with mania, thought control, strange experiences and hallucinations proving non‐significant. 3 and 1 psychotic symptom, but not 2, were associated with counselling/therapy access in the previous year (one symptom OR = 1.74 (1.02, 1.95); two symptoms OR = 2.69 (1.40, 5.15); three symptoms OR = 3.32 (1.90, 5.83)).	**9 stars.** Large representative, random sample. Comparability was reported between responders and non‐responders. Validated measurement tool for PEs. Controlled for sociodemographic and psychopathological correlates of mental health service use. Associations were reported by different levels of service use.
Saha et al. 2013 (Olfson et al., 2002) Australia, mixed.	**Sample source:** 8773 general household dwelling adults aged 18–65. Carried out in 2007 Observational survey design(cross‐sectional). **Comparison/control group:** The comparison group was survey respondents without delusion‐like experiences.	**PEs:** Delusion‐like experiences assessed using the Composite International Diagnostic Interview (CIDI). **Outcomes:** Lifetime use of GP, psychologist, psychiatrist or any other practitioner for mental health reasons. Lifetime psychiatric admission. Lifetime use of medication for MH. Use of vitamins/herbal remedies for mental health reasons.	Study adjusted for age, gender, and then age, gender and comprehensive social demographics. Did not adjust for psychopathology, but restricted sample to people without CIDI comorbidity.	ORs from final model (age, gender etc. adjusted, restricted to people without comorbid disorders) were as follows: seeing GP: 1.88 (1.2, 2.93); any psychiatrist: 0.93 (0.42, 2.07); any psychologist: 1.89 (1.04, 3.44); any practitioner: 1.65 (1.17, 2.32); lifetime admission‐ no results because of low power; lifetime prescription medication use: 1.86 (1.09, 3.16); any vitamin herbal use in the last 2 weeks: 1.32 (0.79, 2.22).	**8 stars.** Large random sample, with comparability between responders and non‐responders discussed, and an adequate response proportion. Validated measurement tool for the ascertainment of PEs. Statistical control for sociodemographic confounders, design control for psychopathology (restricting a sub‐analysis to people without comorbidity). Unclear if mental health service use assessment was blinded.
Nishida et al. (van Nierop et al., 2011), Japan, mixed.	**Sample source:** Cross‐sectional survey of 4894 students in the Mie prefecture, in grades 7,8 and 9 (ages 12,13, and 14). Carried out in July 2006. **Comparison/control group:** People without PEs on the DISC‐C.	**PEs:** Psychotic‐like experiences identified using the Diagnostic Interview Schedule for Children (DISC‐C) **Outcomes:** Dichotomous item on current contact with medical services	The odds ratio for the association between psychotic‐like experiences and contact with medical care was adjusted for GHQ score.	The crude association between reporting any psychotic‐like experience and being in contact with medical services currently was 1.72 (95%CI: 1.49, 1.98). This was attenuated upon adjustment for score on the GHQ, giving an adjusted estimate of 1.45 (95%CI: 1.23, 1.7).	**7 stars.** Large survey with adjustments for concurrent psychopathological symptoms, and a validated tool for the measurement of psychotic‐like experiences. No description of non‐responders, and non‐random sampling.
Olfson et al. (Murphy et al., 2010),USA, urban.	**Sample source:** Non‐representative survey of primary care patients at a general medicine practice in Manhattan, New York. **Comparison/control group:** People who did not report PEs.	**PEs:** Psychotic symptoms were assessed using the Mini International Neuropsychiatric Interview (MINI). **Outcomes:** Respondents were asked a series of questions relating to the receipt of care for an emotional or mental health problem.	Reports chi‐squared statistics and p‐values for the association between psychotic symptoms and psychiatric hospitalization, lifetime and past‐month mental health visits, and lifetime and past‐month use of psychotropic medication.	Used linear and logistic regression to adjust for sociodemographic variables and concurrent DSM disorders. Logistic regressions controlling for various covariates were used to model associations between psychotic symptoms and DSM‐IV disorders, substance use disorders, suicidal ideation, and psychiatric hospitalizations. Associations were found between psychotic symptoms and all markers of mental health service use (all p‐values from chi‐squared = <0.0001).	**7 stars**. Large sample with non‐responders described. Validated measure of psychotic symptoms and measures service use by self‐report.
Werbeloff et al(Kobayashi et al., 2011), Israel, mixed.	**Sample source:** Longitudinal cohort study of Israeli residents, linked to a national psychiatric hospitalization registry. **Comparison/control group:** Those with no PEs.	**PEs:** Psychotic symptoms were assessed using the Psychiatric Epidemiology Research Interview(PERI). **Outcomes:** Hospitalizations were collected using a national hospitalization register for psychiatric admissions.	Associations between the presence of self‐reported psychotic symptoms and later hospitalization for psychotic disorders, and for nonpsychotic disorders, are reported in the form of odds ratios.	The investigators found evidence for the association between psychotic symptoms and admission for both psychotic (OR for weak symptoms = 3.61, 95%CI: 0.78, 16.78), for strong symptoms: 9.54 (95%CI: 1.92, 47.51) and nonpsychotic disorders (OR for weak symptoms: 1.73 (95%CI: 0.58, 5.16), OR for strong symptoms: 2.01 (0.55, 7.27)	8 stars: large sample with prospective design, and direct measurement of service use in the form of hospitalization.

Based on 13 studies. Unless otherwise stated, included studies employed self‐report measures of service use, and used appropriate statistical testing and presentation of results.

### 3.2 Description of included studies

Most studies did not report non‐response proportion, or compare responders to the target population. All studies were conducted in high‐income countries on mainly urban populations. Measures of PEs were in general more carefully validated than measures of mental health service use. Most studies were large, with numbers of participants around 1000; the smallest sample size was 103 (Daalman et al., 2016), and the largest was 11,937 (Barragán et al., 2016). Many studies used a version of the CIDI (Barragán et al., 2016; DeVylder et al., 2014; Gale et al., 2011; Smeets et al., 2013; van Nierop et al., 2011) to ascertain both PEs and mental health service use. One study employed a screening instrument for psychosis (Murphy et al., 2010), and one negative study employed a tool to ascertain self‐reported symptoms of prodromal psychosis (Kobayashi et al., 2011). Other instruments for measurement of PEs were the CAPE (Armando et al., 2012), the DISC‐C(Nishida et al., 2008), the MINI (Olfson et al., 2002), the PERI (Werbeloff et al., 2012), and the Delaunay and Slade Hallucination Scale (Daalman et al., 2016). Nearly all studies measured use of mental health services by self‐report questionnaire. No studies reported costs associated with PEs or directly assessed routine clinical data on service usage, although one study employed hospitalization registers (Werbeloff et al., 2012). One study of mental health service use in people with PEs adopted a case control design, comparing general psychiatric outpatients with a control group (Kobayashi et al., 2011). Four studies (Armando et al., 2010; DeVylder et al., 2014; Murphy et al., 2010; Nishida et al., 2008) adjusted for co‐morbid depression and anxiety. Two follow‐up studies were included, of which one was based only on a hospitalization outcome (Werbeloff et al., 2012), and another on a total sample size of 163 subjects. Five studies reported effect sizes for different types of service use (Barragán et al., 2016; Gale et al., 2011; Murphy et al., 2010; Olfson et al., 2002; Saha et al., 2013).

### 3.3 Qualitative findings for service use in people with self‐reported PEs

Quality assessment agreed between both raters. All studies that found negative (Kobayashi et al., 2011), borderline (Daalman et al., 2016) or mixed (Armando et al., 2012; Murphy et al., 2010) findings for the association between PEs and mental health service use adjusted or accounted for concurrent affective psychopathology. Among the studies with higher quality ratings, DeVylder et al. (2014), (8 quality points) found associations (odds ratios) of greater than 2 between PEs and self‐reported mental health service use in both the lifetime and recently, after taking account of concurrent affective symptoms and substance use. In contrast, Murphy et al. (2010) (9 quality points) reported that only paranoid experiences were associated with using mental health care after all adjustments were made ‐ estimates for mania, thought interferences, strange experiences and hallucinations were no longer sufficiently precise. Kobayashi et al. (2011) (9 quality points), using a clinical definition of service use (comparing clinic attenders with non‐attenders), found no good statistical evidence for an association between service use and a range of different PE scales, after adjustment for depression ratings.

All studies that did not find association between PE and service use also did not make adjustments for concurrent psychopathology. Although Gale et al. (2011) found statistical evidence for a difference in the prevalence of mental health service use among people with PEs compared to those without, they did not report this in the form of an association ‐ for the purposes of this review, we calculated odds ratios based on reported post‐estimation predicted percentages for service use for subjects with one, two or three PEs, compared to subjects with no PEs (reported in Table 1). Smeets et al. (2013) found that mental heath service was associated with both hallucinations and delusions in a random sample of Dutch adults. In another Dutch study, van Nierop et al. (2011) report that people with PEs but without psychotic disorder were nearly twice as likely to report service use for mental health problems. Among the prospective studies, Daalman et al. (2016) found that nearly 40% of people with hallucinations had used services at five year follow up, compared to 12% in a control group, and Werbeloff (2012) reported increased odds of any psychiatric hospitalization in people with psychotic symptoms based on analyses of Israeli hospitalization registry data. Nishida (2008) found that a greater proportion of Japanese students (at grades 7, 8, and 9) who reported PEs were in contact with medical services compared to those who did not.

### 3.4 Meta‐analysis

The overall meta‐analytic estimate, based on adjusted estimates where available, for the association between PEs and service use from random effects analysis was 2.2 (95% confidence interval 1.66,2.91), and based on studies on a total of 73065 subjects.

### 3.5 Tests for publication bias, sensitivity analyses, and tests for heterogeneity

**Figure 3:**
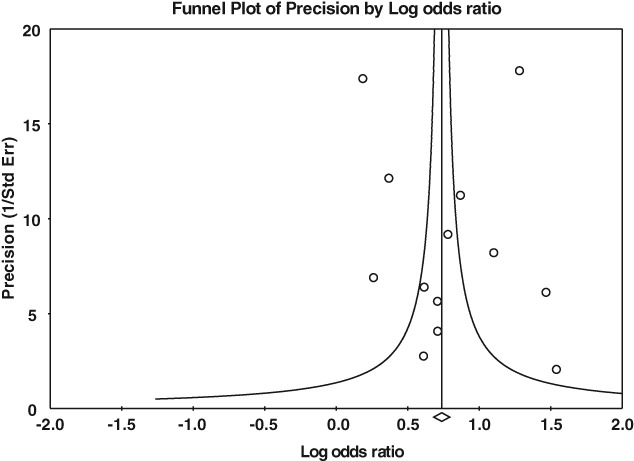
Plot of study precision (1/standard error) against effect size (log odds ratio).

Visual inspection of a funnel plot (Figure 3) revealed no evidence of selective reporting. Quantitative evaluation of risk of publication bias, using a significance test of the Egger regression intercept, corroborated this (intercept = 0.6774, p = 0.80366). The classic fail‐safe procedure suggested 1784 unpublished studies with findings in the opposite direction to the included studies would be necessary to raise the p‐value for the meta‐analytic estimate to above the pre‐specified alpha level. The sensitivity analysis removing one study at a time did not suggest sensitivity of the meta‐analytic estimate to the removal of any one study. Heterogeneity was high, with an I^2^ value of 95.21 and a Q statistic for this model of 250.4 (p < 0.001 on 12 degrees of freedom).

## 4 DISCUSSION

### 4.1 Summary of evidence

To our knowledge, this is the first systematic review addressing the question of whether people reporting PEs use more health services. We found a small number of studies with relatively similar designs, assessing the same underlying construct in different groups of people with PEs, with limited adjustment for alternative explanations for service use, and an emphasis on self‐reported measures of service use. The overall meta‐analytic estimate of the odds ratio, comparing subjects with PEs to those without, was approximately 2, with statistical evidence indicating this was unlikely to be due to chance. Our search returned only two studies that ascertained service use prospectively, one of which only measured hospitalization, and the other had the smallest sample size of all included studies (n = 163). Measurement of PEs was generally better reported than the measurement of service use. Five studies accounted for co‐morbid psychopathology in the association between PEs and mental health service use, finding attenuation of the association when this was done. All studies measured mental health service use through self‐report, with the exception of one case control study which used psychiatric outpatient status as a case definition, and a study based on a hospitalization register.

### 4.2 Limitations of systematic review

Our systematic review had some limitations. We only searched for full text peer‐reviewed articles in English language journals. Health service use is a broad construct, and we may have missed studies meeting our definition because they used slightly different wordings for referring to mental health service use. We were unable to carry out meta‐regression analyses with adequate power to detect the influence of moderating factors, for example the number of PEs reported, the type of PEs, or the type of service use. Included studies were limited to research from high‐income countries, and the structure and availability of health services is highly culturally variant. Although our analysis was based on a large pooled sample size, non‐response was generally poorly described in individual studies.

Meta‐analysis found consistency in the direction of association across studies and statistical evidence for an association between PEs and mental health service use. However statistical heterogeneity was estimated to be high (I^2^ = 95.21), reflecting probable diversity in both the subjects, symptoms, and service use outcomes studies, and in the way studies were designed (Higgins & Green, 2011). Based on the scrutiny of meta‐analytic estimates with one study removed, heterogeneity did not appear to be related to the inclusion/exclusion of any one particular study in this review. Moreover, only a minority of studies adjusted for confounders or reported sample non‐response in detail, leading to a risk of bias. All studies that adjusted for concurrent psychopathology showed weaker associations and weaker evidence for an association than studies that did not adjust for concurrent psychopathology. Despite high heterogeneity, there was little evidence of publication bias based on inspection of the funnel plot (Figure 3). Finally, there were only two prospective studies included in this review, limiting inferences about the direction of the association between PEs and use of mental health care.

### 4.3 Explanations

Although people with PEs may be more likely to use, or have used, mental health services compared to the general population, the factors which result in help‐seeking in this group remain to be determined. The findings of this review could be due to a direct effect of PEs on service use, or due to confounding, that is, PEs and service use could both result from other factors. For example, our results suggest that concurrent psychopathology, such as anxiety and depression, could play a key role in determining service use, although possibly with an independent role for PEs themselves. However, all studies that adjusted for concurrent psychopathology reported attenuated associations with mental health service use. Thus, our results are also consistent with a residual confounding explanation, that is, that improved measurement of concurrent psychopathology in people with PEs in these studies might have removed the association completely, and therefore that the associations with service use demonstrated in these studies might be accounted for by depression and other psychopathology rather than attributable to PEs in themselves. The weaker estimates found in studies that adjusted for concurrent affective symptoms are also consistent with mediation of the effect of PEs on service use by affective symptoms. Based on the findings of this review, more studies with longitudinal designs are needed to advance this question. Psychotic experiences are strongly related to suicidal thinking or behaviour (Kelleher et al., 2013; Kelleher et al., 2014a), and this relationship might also explain increased use of healthcare in people with PEs.

Although evaluation of service use is a complex concept (Gladstone et al., 2007), measurement of the details of use of services in these studies was generally very limited and varied between studies. There is evidence that self‐reported service use is liable to recall bias, and other forms of systematic measurement error (Rhodes & Fung, 2004; Rhodes et al., 2002).

Very few if any studies measured service use following the onset or demonstration of PEs. This is important because if PEs are to be a focus for primary prevention, then we must discern whether PEs are really an early marker for later service use, or occur concurrently with other psychopathology once diagnoses have already been made/help has been sought. Statistical adjustment is only one, albeit easily performed approach to accounting for the effects of other psychopathology on the measured effect‐ other approaches would be to match participants on the concurrent psychopathology, or restrict the sample to subjects without any other mental health problem.

### 4.4 Implications

There is now consensus that although the majority of people with PEs remain well, they are a general indicator of diagnosable psychopathology (McGorry et al., 2008; Yung et al., 2008). Indeed, research from a range of centres now indicates that PEs are a marker for a broad range of adverse outcomes, ranging from poor mental health (Saha et al., 2011a), physical health (Saha et al., 2011b), social and occupational functioning (Kelleher et al., 2014b), drug and alcohol use problems (Saha et al., 2011c), trauma (Morgan et al., 2014), social disadvantage (Morgan et al., 2014), and social isolation (Saha et al., 2012). The present study demonstrates the possible relevance of these impairments for the planning of mental health services, and the need for well designed studies on this topic. The work summarised here clearly indicates that PEs could be relevant for the indexing of a range of mental health problems, not only psychosis risk (Van Os et al., 2009). Moreover, PEs could be a reliable indicator of later need for health care, even if they are not causally related to that need; screening for PEs in primary care might be of value in facilitating prompt intervention in a sub‐group at elevated risk of requiring secondary mental health care.

The absolute increase in psychosis risk attributable to psychotic symptoms is low. For example, in the meta‐analysis by Kaymaz et al. (2012), the incidence rate of psychosis in those with ascertained PEs was 558 per 100 000 person years of follow up (0.6% per year), compared to 159 cases per 100 000 person‐years in those without (0.2% per year). Transitions to psychosis are much higher in enriched clinical samples, for example a meta‐analysis of transition occurrence in ultra‐high risk samples suggested an overall transition risk of 36% at three years follow up (Fusar‐Poli et al., 2012). This lends support to the ultra‐high risk approach, but also is consistent with a model where PEs represent a general marker for more severe psychopathology. The present review has demonstrated a consistent, but methodologically weak association with health service use in this population, and suggests that PEs may mark a considerable unmet mental health need in the general population.

### 4.5 Future research

Many individuals who later develop psychosis experience a long duration of untreated symptoms, and not all who experience prodromal symptoms seek help (O'Callaghan et al., 2010; Wang et al., 2005). Aside from personal barriers to access to care, such as concerns about privacy, and financial/time constraints (Eisenberg et al., 2007; Hunt & Eisenberg, 2010), there are likely to be important symptomatic and demographic predictors of service use in people with PEs. Investigating these associations requires accurate measures of mental health service use, across a broad range of settings. For example, recall bias in the evaluation of recent service use might be limited by shorter time periods of recall, or triangulation of self‐report information with linked electronic health records from care providers (Stewart et al., 2009).

Depressive symptoms are common in people with PEs, and are associated with distress and need for care (Yung et al., 2007). If the association between PEs and service use is accounted for by concurrent psychopathology, and is not really causal, then intervening on PEs will not reduce use of services in people with PEs. Therefore, more detailed assessment of the role played by depressive and other mental health symptoms on use of mental health services is necessary in order to build good evidence for early intervention. In particular, PEs can be accompanied by a broad range of symptoms beyond only depressive symptoms (Kelleher et al., 2014a), suggesting that proper understanding of the relationship between PEs and use of healthcare will require measurement of a fuller range of the psychopathology that might be present with PEs.

Finally, assessing the role that PEs might play in prevention of severe mental disorders requires clarity on the temporal relationship between PEs, the existence of diagnosable mental disorder, and pathways into mental health service use. This is particularly important in light of the probably transitory nature of most PEs (Chapman et al., 1994; Hanssen et al., 2005).

### 4.6 Conclusions

This review identified some PE research suggesting that people who report PEs also report more service use, and some studies report that this is true when other forms of psychopathology are adjusted for. However, in order to establish that PEs lead to service uses, irrespective of diagnosis, better studies are required. In particular studies are needed which do not rely on the self report of service use, and which are sensitive to the timing of service use and of the onset of PEs (Yung et al., 2012).

## Supporting information


**Appendix S1.** Full electronic search strategy.

## References

[eip12464-bib-0001] Armando, M. , Nelson, B. , Yung, A. R. , et al. (2010). Psychotic‐like experiences and correlation with distress and depressive symptoms in a community sample of adolescents and young adults. Schizophrenia Research, 119, 258–265. 20347272 10.1016/j.schres.2010.03.001

[eip12464-bib-0002] Armando, M. , Nelson, B. , Yung, A. R. , et al. (2012). Psychotic experience subtypes, poor mental health status and help‐seeking behaviour in a community sample of young adults. Early Intervention in Psychiatry, 6, 300–308. 22029711 10.1111/j.1751-7893.2011.00303.x

[eip12464-bib-0003] American Psychiatric Association . (2013). Diagnostic and statistical manual of mental disorders (5th ed.). Arlington, VA: American Psychiatric Publishing.

[eip12464-bib-0004] Barragán, A. , Yamada, A.‐M. , Lee, K. K. , & Barrio, C. (2016). Correlates in the Endorsement of Psychotic Symptoms and Services Use: Findings from the Collaborative Psychiatric Epidemiology Surveys. Community Mental Health Journal, 52, 631–642. 25693679 10.1007/s10597-015-9850-z

[eip12464-bib-0056] Biostat. Inc. (2014). *Comprehensive Meta Analysis* (Version 3.3.070). Biostat Inc. Englewood, NJ. USA. Retrieved from https://www.meta-analysis.com.

[eip12464-bib-0005] Chapman, L. J. , Chapman, J. P. , Kwapil, T. R. , Eckblad, M. , & Zinser, M. C. (1994). Putatively psychosis‐prone subjects 10 years later. Journal of Abnormal Psychology, 103, 171. 8040487 10.1037//0021-843x.103.2.171

[eip12464-bib-0006] Daalman, K. , Diederen, K. , Hoekema, L. , van Lutterveld, R. , & Sommer, I. (2016). Five year follow‐up of non‐psychotic adults with frequent auditory verbal hallucinations: are they still healthy? Psychological Medicine, 46(9), 1897–1907. 26961499 10.1017/S0033291716000386

[eip12464-bib-0007] DerSimonian, R. , & Laird, N. (1986). Meta‐analysis in clinical trials. Controlled Clinical Trials, 7, 177–188. 3802833 10.1016/0197-2456(86)90046-2

[eip12464-bib-0008] DeVylder, J. E. , HY, O. , Corcoran, C. M. , & Lukens, E. P. (2014). Treatment seeking and unmet need for care among persons reporting psychosis‐like experiences. Psychiatric Services, 65(6), 774–780. 24534875 10.1176/appi.ps.201300254 PMC6483726

[eip12464-bib-0009] Egger, M. , Smith, G. D. , Schneider, M. , & Minder, C. (1997). Bias in meta‐analysis detected by a simple, graphical test. British Medical Journal, 315, 629–634. 9310563 10.1136/bmj.315.7109.629 PMC2127453

[eip12464-bib-0010] Eisenberg, D. , Golberstein, E. , & Gollust, S. E. (2007). Help‐seeking and access to mental health care in a university student population. Medical Care, 45, 594–601. 17571007 10.1097/MLR.0b013e31803bb4c1

[eip12464-bib-0011] Fusar‐Poli, P. , Bonoldi, I. , Yung, A. R. , et al. (2012). Predicting psychosis: meta‐analysis of transition outcomes in individuals at high clinical risk. Archives of General Psychiatry, 69, 220–229. 22393215 10.1001/archgenpsychiatry.2011.1472

[eip12464-bib-0012] Fusar‐Poli, P. , Borgwardt, S. , Bechdolf, A. , et al. (2013). The psychosis high‐risk state: a comprehensive state‐of‐the‐art review. JAMA Psychiatry, 70, 107–120. 23165428 10.1001/jamapsychiatry.2013.269 PMC4356506

[eip12464-bib-0013] Fusar‐Poli, P. , Nelson, B. , Valmaggia, L. , Yung, A. R. , & McGuire, P. K. (2014). Comorbid depressive and anxiety disorders in 509 individuals with an at‐risk mental state: impact on psychopathology and transition to psychosis. Schizophrenia Bulletin, 40, 120–131. 23180756 10.1093/schbul/sbs136 PMC3885287

[eip12464-bib-0014] Gale, C. , Wells, J. , McGee, M. , & Browne, O. (2011). A latent class analysis of psychosis‐like experiences in the New Zealand Mental Health Survey. Acta Psychiatrica Scandinavica, 124, 205–213. 21495982 10.1111/j.1600-0447.2011.01707.x

[eip12464-bib-0015] Gladstone, B. M. , Volpe, T. , & Boydell, K. M. (2007). Issues encountered in a qualitative secondary analysis of help‐seeking in the prodrome to psychosis. The Journal of Behavioral Health Services & Research, 34, 431–442. 17694437 10.1007/s11414-007-9079-x

[eip12464-bib-0016] Hanssen, M. , Bak, M. , Bijl, R. , Vollebergh, W. , & Os, J. (2005). The incidence and outcome of subclinical psychotic experiences in the general population. British Journal of Clinical Psychology, 44, 181–191. 16004653 10.1348/014466505X29611

[eip12464-bib-0017] Higgins, J. P. , & Green, S. (2011). Cochrane handbook for systematic reviews of interventions. Chichester, England: John Wiley & Sons.

[eip12464-bib-0018] Hunt, J. , & Eisenberg, D. (2010). Mental health problems and help‐seeking behavior among college students. Journal of Adolescent Health, 46, 3–10. 20123251 10.1016/j.jadohealth.2009.08.008

[eip12464-bib-0020] Kaymaz, N. , Drukker, M. , Lieb, R. , et al. (2012). Do subthreshold psychotic experiences predict clinical outcomes in unselected non‐help‐seeking population‐based samples? A systematic review and meta‐analysis, enriched with new results. Psychological Medicine, 42, 2239–2253. 22260930 10.1017/S0033291711002911

[eip12464-bib-0021] Kelleher, I. , Connor, D. , Clarke, M. C. , Devlin, N. , Harley, M. , & Cannon, M. (2012b). Prevalence of psychotic symptoms in childhood and adolescence: a systematic review and meta‐analysis of population‐based studies. Psychological Medicine, 42, 1857–1863. 22225730 10.1017/S0033291711002960

[eip12464-bib-0022] Kelleher, I. , Corcoran, P. , Keeley, H. , et al. (2013). Childhood trauma and psychotic experiences‐cause, effect and directionality: Results from a prospective cohort study. European Child and Adolescent Psychiatry, 1, S200.

[eip12464-bib-0023] Kelleher, I. , Devlin, N. , Wigman, J. T. , et al. (2014a). Psychotic experiences in a mental health clinic sample: implications for suicidality, multimorbidity and functioning. Psychological Medicine, 44, 1615–1624. 24025687 10.1017/S0033291713002122

[eip12464-bib-0024] Kelleher, I. , Devlin, N. , Wigman, J. T. W. , et al. (2014b). Psychotic experiences in a mental health clinic sample: implications for suicidality, multimorbidity and functioning. Psychological Medicine, 44, 1615–1624. 24025687 10.1017/S0033291713002122

[eip12464-bib-0025] Kelleher, I. , Lynch, F. , Harley, M. , et al. (2012a). Psychotic symptoms in adolescence index risk for suicidal behavior: Findings from 2 population‐based case‐control clinical interview studies. Archives of General Psychiatry, 69, 1277–1283. 23108974 10.1001/archgenpsychiatry.2012.164

[eip12464-bib-0026] Kobayashi, H. , Nemoto, T. , Murakami, M. , Kashima, H. , & Mizuno, M. (2011). Lack of association between psychosis‐like experiences and seeking help from professionals: A case‐controlled study. Schizophrenia Research, 132, 208–212. 21865013 10.1016/j.schres.2011.07.029

[eip12464-bib-0027] Linscott, R. J. , & van Os, J. (2013a). An updated and conservative systematic review and meta‐analysis of epidemiological evidence on psychotic experiences in children and adults: on the pathway from proneness to persistence to dimensional expression across mental disorders. Psychological Medicine, 43, 1133–49. 22850401 10.1017/S0033291712001626

[eip12464-bib-0028] Linscott, R. J. , & van Os, J. (2013b). An updated and conservative systematic review and meta‐analysis of epidemiological evidence on psychotic experiences in children and adults: on the pathway from proneness to persistence to dimensional expression across mental disorders. Psychological Medicine, 43, 1133–49. 22850401 10.1017/S0033291712001626

[eip12464-bib-0029] McGorry, P. D. , Killackey, E. , & Yung, A. (2008). Early intervention in psychosis: concepts, evidence and future directions. World Psychiatry, 7, 148–156. 18836582 10.1002/j.2051-5545.2008.tb00182.x PMC2559918

[eip12464-bib-0030] Moher, D. , Liberati, A. , Tetzlaff, J. , & Altman, D. G. (2009). Preferred reporting items for systematic reviews and meta‐analyses: the PRISMA statement. Annals of Internal Medicine, 151, 264–269. 19622511 10.7326/0003-4819-151-4-200908180-00135

[eip12464-bib-0031] Morgan, C. , Reininghaus, U. , Reichenberg, A. , Frissa, S. , Hotopf, M. , & Hatch, S. L. (2014). Adversity, cannabis use and psychotic experiences: evidence of cumulative and synergistic effects. The British Journal of Psychiatry, 204, 346–353. 24627297 10.1192/bjp.bp.113.134452 PMC4006086

[eip12464-bib-0032] Murphy, J. , Shevlin, M. , Houston, J. , & Adamson, G. (2010). A Population Based Analysis of Subclinical Psychosis and Help‐Seeking Behavior. Schizophrenia Bulletin, 38(2), 360‐367. 20709763 10.1093/schbul/sbq092 PMC3283148

[eip12464-bib-0033] Nishida, A. , Tanii, H. , Nishimura, Y. , et al. (2008). Associations between psychotic‐like experiences and mental health status and other psychopathologies among Japanese early teens. Schizophrenia Research, 99, 125–133. 18248792 10.1016/j.schres.2007.11.038

[eip12464-bib-0034] O'Callaghan, E. , Turner, N. , Renwick, L. , et al. (2010). First episode psychosis and the trail to secondary care: help‐seeking and health‐system delays. Social Psychiatry and Psychiatric Epidemiology, 45, 381–391. 19578801 10.1007/s00127-009-0081-x

[eip12464-bib-0035] Olfson, M. , Lewis‐Fernández, R. , Weissman, M. M. , et al. (2002). Psychotic symptoms in an urban general medicine practice. American Journal of Psychiatry, 159, 1412–1419. 12153836 10.1176/appi.ajp.159.8.1412

[eip12464-bib-0036] Rhodes, A. E. , & Fung, K. (2004). Self‐reported use of mental health services versus administrative records: care to recall? International Journal of Methods in Psychiatric Research, 13, 165–175. 15297900 10.1002/mpr.172 PMC6878470

[eip12464-bib-0037] Rhodes, A. E. , Lin, E. , & Mustard, C. A. (2002). Self‐reported use of mental health services versus administrative records: should we care? International Journal of Methods in Psychiatric Research, 11, 125–133. 12459825 10.1002/mpr.130 PMC6878364

[eip12464-bib-0038] Rothstein, H. R. , Sutton, A. J. , & Borenstein, M. (2006). Publication bias in meta‐analysis: Prevention, assessment and adjustments. Hoboken, NJ. USA: John Wiley & Sons.

[eip12464-bib-0039] Saha, S. , McGrath, J. , & Scott, J. (2013). Service Use for Mental Health Problems in People with Delusional‐Like Experiences: A Nationwide Population Based Survey. PloS One, 8(8), e71951. https://doi:10.1371/journal.pone.0071951 10.1371/journal.pone.0071951 PMC3749219 23991012

[eip12464-bib-0040] Saha, S. , Scott, J. , Varghese, D. , & McGrath, J. (2011b). The association between physical health and delusional‐like experiences: a general population study. PloS One, 6(4), e18566. https://doi:10.1371/journal.pone.0018566 10.1371/journal.pone.0018566 PMC3081831 21541344

[eip12464-bib-0041] Saha, S. , Scott, J. , Varghese, D. , & McGrath, J. (2012). Social support and delusional‐like experiences: a nationwide population‐based study. Epidemiology and Psychiatric Sciences, 21, 203–212. 22789170 10.1017/S2045796011000862

[eip12464-bib-0042] Saha, S. , Scott, J. G. , Varghese, D. , Degenhardt, L. , Slade, T. , & McGrath, J. J. (2011c). The association between delusional‐like experiences, and tobacco, alcohol or cannabis use: a nationwide population‐based survey. BMC Psychiatry, 11(1), 202. 22204498 10.1186/1471-244X-11-202 PMC3313864

[eip12464-bib-0043] Saha, S. , Scott, J. G. , Varghese, D. , & McGrath, J. J. (2011a). The association between general psychological distress and delusional‐like experiences: a large population‐based study. Schizophrenia Research, 127, 246–251. 21239145 10.1016/j.schres.2010.12.012

[eip12464-bib-0044] Smeets, F. , Lataster, T. , Van Winkel, R. , De Graaf, R. , Ten Have, M. , & Van Os, J. (2013). Testing the hypothesis that psychotic illness begins when subthreshold hallucinations combine with delusional ideation. Acta Psychiatrica Scandinavica, 127, 34–47. 22676336 10.1111/j.1600-0447.2012.01888.x

[eip12464-bib-0045] Stewart, R. , Soremekun, M. , Perera, G. , et al. (2009). The South London and Maudsley NHS foundation trust biomedical research centre (SLAM BRC) case register: development and descriptive data. BMC Psychiatry, 9, 51. 19674459 10.1186/1471-244X-9-51 PMC2736946

[eip12464-bib-0046] van Nierop, M. , van Os, J. , Gunther, N. , et al. (2011). Phenotypically continuous with clinical psychosis, discontinuous in need for care: evidence for an extended psychosis phenotype. Schizophrenia Bulletin, 38(2), 231‐238. 21908795 10.1093/schbul/sbr129 PMC3283149

[eip12464-bib-0047] Van Os, J. , Linscott, R. J. , Myin‐Germeys, I. , Delespaul, P. , & Krabbendam, L. (2009). A systematic review and meta‐analysis of the psychosis continuum: evidence for a psychosis proneness–persistence–impairment model of psychotic disorder. Psychological Medicine, 39, 179–95. 18606047 10.1017/S0033291708003814

[eip12464-bib-0048] Wang, P. S. , Berglund, P. , Olfson, M. , Pincus, H. A. , Wells, K. B. , & Kessler, R. C. (2005). Failure and delay in initial treatment contact after first onset of mental disorders in the National Comorbidity Survey Replication. Archives of General Psychiatry, 62, 603–613. 15939838 10.1001/archpsyc.62.6.603

[eip12464-bib-0049] Wells, G. , Shea, B. , O'connell, D. , et al. (2000). The Newcastle‐Ottawa Scale (NOS) for assessing the quality of nonrandomised studies in meta‐analyses. Department of Epidemiology and Community Medicine, University of Ottawa, Canada University of Ottawa, Canada: Available at: http://www.ohri.ca/programs/clinical_epidemiology/oxford.asp

[eip12464-bib-0050] Werbeloff, N. , Drukker, M. , Dohrenwend, B. P. , et al. (2012). Self‐reported attenuated psychotic symptoms as forerunners of severe mental disorders later in life. Archives of General Psychiatry, 69, 467–475. 22213772 10.1001/archgenpsychiatry.2011.1580

[eip12464-bib-0051] World Health Organization (1992). The ICD‐10 classification of mental and behavioural disorders: clinical descriptions and diagnostic guidelines. Geneva, Switzerland: World Health Organization.

[eip12464-bib-0052] Yung, A. R. , Buckby, J. A. , Cosgrave, E. M. , et al. (2007). Association between psychotic experiences and depression in a clinical sample over 6 months. Schizophrenia Research, 91, 246–253. 17239566 10.1016/j.schres.2006.11.026

[eip12464-bib-0053] Yung, A. R. , Buckby, J. A. , Cotton, S. M. , et al. (2006). Psychotic‐like experiences in nonpsychotic help‐seekers: associations with distress, depression, and disability. Schizophrenia Bulletin, 32, 352–359. 16254060 10.1093/schbul/sbj018 PMC2632224

[eip12464-bib-0054] Yung, A. R. , Nelson, B. , Stanford, C. , et al. (2008). Validation of “prodromal” criteria to detect individuals at ultra high risk of psychosis: 2 year follow‐up. Schizophrenia Research, 105, 10–17. 18765167 10.1016/j.schres.2008.07.012

[eip12464-bib-0055] Yung, A. R. , Woods, S. W. , Ruhrmann, S. , et al. (2012). Whither the attenuated psychosis syndrome? Schizophrenia Bulletin, 38, 1130–1134. 23144056 10.1093/schbul/sbs108 PMC3494060

